# Green Approach Extraction of Perezone from the Roots of *Acourtia platyphilla* (A. Grey): A Comparison of Four Activating Modes and Supercritical Carbon Dioxide

**DOI:** 10.3390/molecules24173035

**Published:** 2019-08-21

**Authors:** René Escobedo-González, Andrea Vázquez Vázquez Cabañas, Armando Martínez González, Pablo Mendoza Sánchez, Zenaida Saavedra-Leos, Julián Cruz-Olivares, Juan Nava Serrano, Joel Martínez, René Miranda Ruvalcaba

**Affiliations:** 1 Department of industrial maintenance and nanotechnology, Technological University of Juarez City, Ciudad Juarez, Chihuahua 32695, Mexico; 2 Department of Chemistry, Faculty of Superior Studies Cuautitlan, Campus 1, Autonomous National University of Mexico, Cuautitlan Izcalli, Mexico State 54740, Mexico; 3 Academic Coordination, Altiplano Region, Autonomous University of San Luis Potosi, Road Cedral km. 5+600, Matehuala, San Luis Potosi 78700, Mexico; 4 Chemistry Faculty, Autonomous University of Mexico State, Toluca, State of Mexico 501020, Mexico; 5 Biological Science National School, National Polytechnic Institute, Mexico City 11340, Mexico; 6 Chemistry Science Faculty, Autonomous University of San Luis, Av. Dr. Manuel Nava 6, State of San Luis Potosi 78210, Mexico

**Keywords:** green approach, perezone-sesquiterpene quinone, comparative extraction, microwaves, infrared, ultrasound, supercritical carbon dioxide

## Abstract

Perezone, a sesquiterpene quinone, is a very important molecule due to its pharmacological activities in addition to the fact that it is considered to be the first secondary metabolite isolated in the new world (America–Mexico, 1852). This study aims to offer a green comparative study about the extraction of the target molecule from the roots of the vegetable specimen *Acourtia platyphilla* (A. Grey). The study was performed comparing five different modes of extraction: supercritical CO_2_, electromagnetic infrared and microwave irradiations, mechanical-wave ultrasound versus typical mantle heating procedure. An exhaustive comparative-discussion of the obtained results is provided. It is worth noting that the corresponding quantifications were established using ^1^H NMR, correlating appropriately the integrals of the vinylic proton H-6 of perezone with the aromatic singlet of *p*-dinitrobenzene employed as an internal reference. It is also important to highlight that the four presented procedures are novel modes to extract perezone. Finally, a complementary study about the solubility of the target sesquiterpene quinone related to the use of supercritical CO_2_ is also reported.

## 1. Introduction

**Figure 1:**
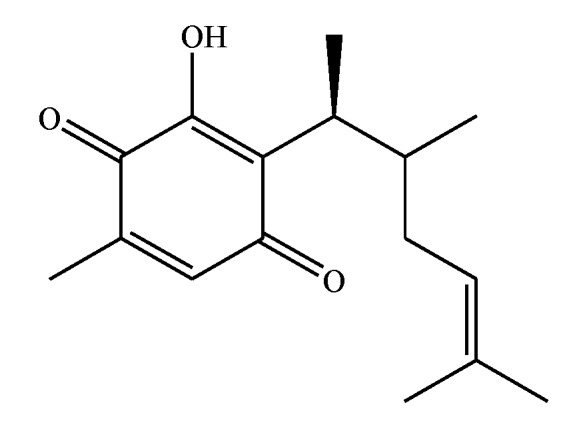
Perezone.

Many quinones are interesting secondary metabolites which are mainly isolated from plants [1], in particular, for this study perezone or pipitzaoic acid, a sesquiterpene quinone (Figure 1), is recognized as the first secondary metabolite isolated on the American continent (New World) by Leopoldo Río de la Loza [2] from the roots of *Perezia* (actually *Acourtia*) specimens. In 1856, Dr. de la Loza received a gold medal for his chemical discoveries from the Society for the Protection of Industrial Arts in London. This important molecule has been the target of many chemical, structural, and biological studies, e.g., its reactivity into pipitzols [3,4,5], its transformation into isoperezone [6], several green contributions [7,8], structural elucidation (NMR studies) [9,10,11]. It is important to note that it has been used as a pigment [9] highlighting several pharmacological effects [12,13,14,15], such as the release [16] of mitochondrial Ca^2+^, and finally, the ability of some of its derivatives to produce cytotoxic activity [17,18,19].

Since its inception in the early 1990s, green chemistry, a field of actuality, has focused on the molecular level to accomplish sustainability. It is now positioned as an important scientific discipline to prevent pollution. This class of chemistry has a code of behavior known as the Twelve Principles [20]. In this respect, chemical researchers must design innovative processes which present low or no risk to the environment. It is worth noting that no chemical activity is entirely risk- and waste-free; therefore, the “Twelve Principles of Green Chemistry” need to be understood and managed as a scientific reflection [21]. For example, most chemical processes employ thermal sources created from fossil fuels, and only a few of them are devised from biomass and non-carbon sources [22]. As a complement to the above mentioned, “Principle 6” of the Green Chemistry Protocol (minimize energy requirements) should be recognized due to its environmental and economic impacts. Hence, from a green chemistry perspective, attempts must be undertaken to make the energy input in chemical systems as efficient as possible. Valuable methods have been developed as alternative modes to the classical mode, known as mantle heating (MH). They are a) the mechanical modes sonication (US) and tribochemistry-mechanical milling (MM), and b) several electromagnetic irradiation modes, e.g., microwave (MW), infrared (IR), and electrochemistry [23]. All are employed to minimize reaction time, improve the product yield, and avoid undesired by-products.

In a recent review and a most recent book chapter [24,25], our research group provided interesting and complete information related to the use of infrared irradiation in its three zones (NIR: near-infrared, MIR: middle infrared, and FIR: far infrared) as a clean and effective mode to activate a reaction, as well as an appropriate procedure for the extraction of natural products. In addition, the extraction of essential oils and secondary metabolites using supercritical fluid technology is an alternative process [26,27,28,29]. In particular, the use of supercritical carbon dioxide (scCO_2_) instead of traditional chemical solvents, is more convenient since the removal and the purification of solvents is avoided improving the quality of the obtained products [30,31]. Unfortunately, there is little information available on the solubility of natural compounds in scCO_2_. In this sense, to enhance the selectivity of an extraction process, knowledge about the solubility of the component to be extracted by the supercritical fluid is required.

In recent years, cancer prevention using natural products has received considerable attention; consequently, the chemistry of secondary metabolites is a powerful source for novel drug candidates. The design and implementation of synthetic processes moving toward the Green Chemistry Protocol [32,33,34,35,36] is one of the major challenges in modern organic synthesis.

As a role of our up-to-date research program, we are currently carrying out green synthetic strategies to synergize or modify the pharmacological activities of single known compounds by constructing novel hybrid molecules, using non-conventional activating sources, such as MW and NIR irradiations, the mechanical modes US and MM, in the absence of solvents or using innocuous solvents [7,37,38,39,40,41,42,43].

Taking into account the aforementioned commentaries, this study aims to (a) achieve a green contribution to the chemistry of the first secondary metabolite isolated in the new world; (b) offer a green comparative study about its extraction from the roots of *Acourtia platyphilla*, evaluating five different modes of extraction, using: scCO_2_, MW, NIR, and US versus the typical MH; and (c) make an evaluation about the solubility of perezone in supercritical carbon dioxide at different pressure conditions to find appropriate conditions for the extraction of perezone.

## 2. Results and Discussion

**Figure 2:**
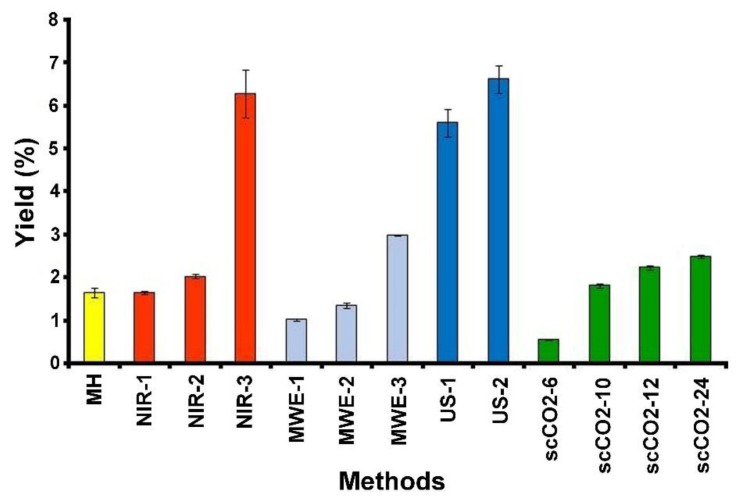
Comparative yield extraction percent of perezone for the different treatments employed.

**Figure 3:**
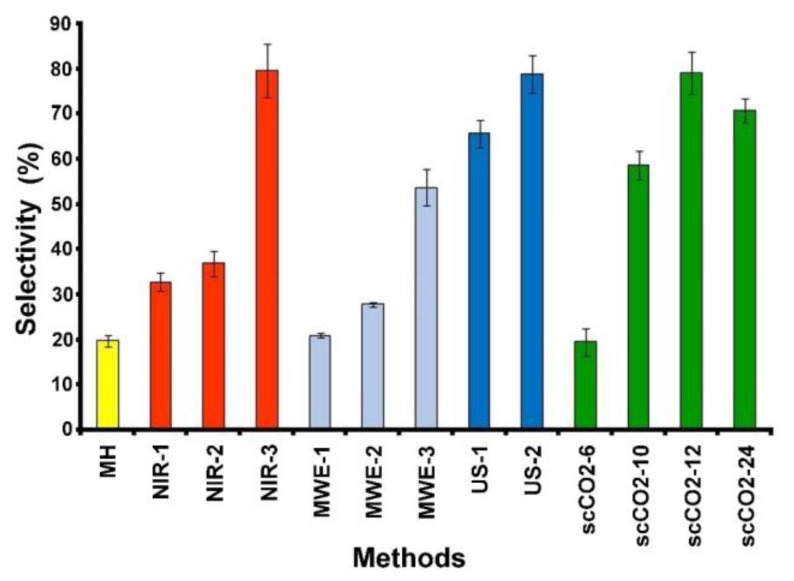
Comparative selectivity extraction percent of perezone for the different treatments employed.

**Figure 4:**
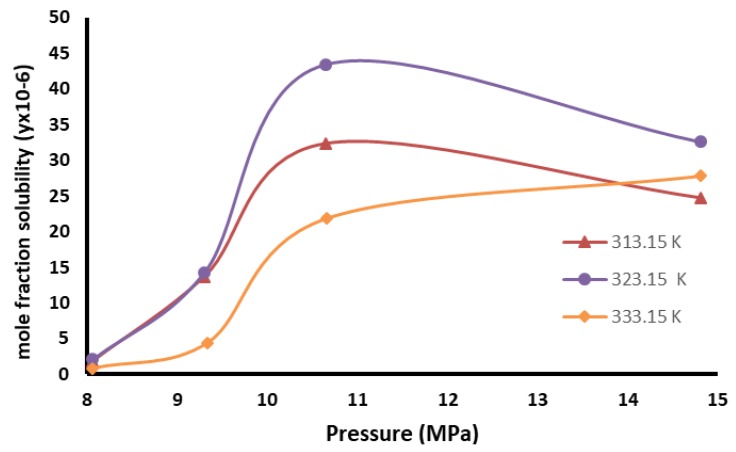
Perezone solubility isotherms in scCO_2_.

**Figure 5:**
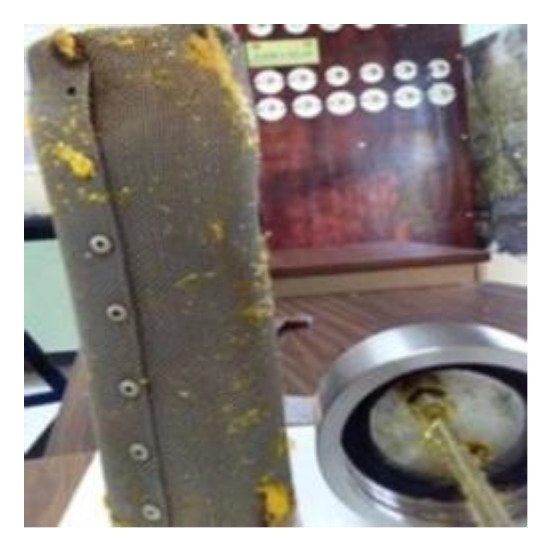
Perezone extraction with scCO_2_, the yellow crystals correspond to the isolated product.

The obtained results are summarized in Figure 2, Figure 3, Figure 4 and Figure 5 and Table 1.

### 2.1. Conventional Thermal Extraction

Conventional perezone thermal extraction from the vegetal species roots was performed in agreement with previous reports [44,45]. These results were used as a reference for yield and selectivity.

The yield of perezone from the roots of *A. platyphilla* (1.652%), Figure 2, agrees with reported values [44,45]. Regarding selectivity, the conventional process reveals a value of 19.784%, Figure 3.

### 2.2. Near-Infrared Promoted Extraction

The near-infrared-assisted extraction was evaluated using three different conditions: (1) 5 g of dried and ground roots were irradiated in the absence of solvent during 15 min at 121 °C (the minimal permitted), these conditions are referred to as NIR-1; (2) 5g of dried and ground roots were irradiated for 15 min with NIR in the presence of solvent (*n*-hexane, 30 mL), the total irradiation time was 15 min, which was divided into intervals of 5 min, with stand-by periods between these intervals of 5 min (NIR-2); and finally (3) 5 g of dried roots were irradiated in the presence of 30 mL of *n*-hexane, during a total period of 30 min, divided into intervals of 5 min of irradiation followed of 5 min without irradiation to complete the total time (NIR-3), (Materials and Methods section). Obtained yield and selectivity values are shown in Figure 2 and Figure 3, respectively.

According to the results, in the case of NIR-1, perezone was detected with an average yield of 1.656 ± 0.02%, a value statically equal to that previously obtained by the conventional extraction. In addition, the corresponding selectivity parameter was 32.745% in comparison to 19.784% for conventional conditions.

On the other hand, NIR-2 showed a perezone yield of 2.036%, exhibiting a statistical difference to the NIR-1 method of 1.656% and 1.652% for the conventional process.

The highest yield of perezone extraction was achieved under NIR-3 conditions, with an increment of 400% (6.27%) compared with that obtained by the conventional mantle heating methodology (1.652%).

The yield differences among the conventional thermal process and the NIR extractions can be attributed to the fact that infrared irradiation is directly and easily absorbed by the root-tissues, and consequently, provides more efficient heating. In other words, infrared irradiation is a straight heating mode. Infrared irradiation promotes vibrational modes in a molecule providing high energy efficiency, activating a reaction, but more importantly, it favors a metabolite extraction [24,25] which is the target of this work.

### 2.3. Microwave Promoted Extraction

The perezone microwave promoted extraction (MWE), 5 g of root sample and 30 mL of *n*-hexane as a solvent, was carried out varying the temperature, the microwave power, and the contact time with the microwave irradiation (Materials and Methods section). The first probe (MWE-1) parameters were 100 W, 50 °C, 5 min duration. The results showed a significant difference to the conventional heating mode, with a lower yield of perezone (1.027%), Figure 2.

The second test (MWE-2) involved both a decrease of the microwave irradiation power and the exposure time (30 W, 50 °C, 3.5 min), but the same temperature. These results showed a yield of 1.353%. The value has a significant difference to the reference procedure (conventional) as it is lower. The MWE-2 results showed an increase in yield and selectivity in comparison with MWE-1 with significant differences.

For the last evaluation in this microwave irradiations set, (MWE-3), the parameters were 100 W, 60 °C, 10 min duration. The obtained results demonstrated an increase in the perezone yield higher in comparison with the conventional procedure (2.995%), Figure 2.

MWE has been widely employed for many other procedures [46,47,48,49,50]. However, it is important to highlight that this methodology is highly dependent on the nature of the solvent. A more polar solvent (major dielectric constant) generally achieves more interaction with microwave irradiation [51]. Since the hexane is a less polar solvent, it has less interaction with the microwaves and less efficiency. The yields obtained by MWE were similar to the conventional method; however, these were smaller compared with infrared assisted extraction despite both are activated by electromagnetic waves.

### 2.4. Ultrasound Promoted Extraction

Related to the ultrasound study, 5 g of the specimen roots with 30 mL of *n*-hexane as solvent (Materials and Methods section), there were minimal variations: exposition time (30 and 60 min) with constant temperature (60 °C) and constant 42 kHz frequency. Figure 2 and Figure 3 summarize the corresponding results in percent for yield and selectivity.

The ultrasound-assisted extraction for a duration of 30 min (US-1) afforded a higher yield (5.609%), with a significant difference to the conventional method, 350% more than conventional activation. Regarding the selectivity of this methodology, it increased (65.708%) in comparison with the mantle heating process (19.784%). Moreover, increasing the time of ultrasound treatment to 60 min (US-2) improved the extraction yield (6.623%), a value corresponding to nearly 400% of the amount obtained in the MH procedure.

Regarding the ultrasound-assisted extraction it is important to note that this type of mechanical wave activation generates a cavitation effect, producing efficient interaction with the cellular structure in several tissues, breaking-off the cellular membrane, allowing the solvent penetration and the release of the intracellular content [52,53,54,55,56].

### 2.5. Supercritical Dioxide Perezone Extraction

The supercritical carbon dioxide is a selective and green solvent, which can modify its properties with the change in the work pressure and temperature. For the perezone extraction, the first step was developing a solubility study of perezone in scCO_2_. Figure 4 shows the perezone solubility isotherms in scCO_2_ using a pressure range 8 to 15 MPa and temperatures of 313.15, 323.15, and 333.15 K.

The graph shows an increase in solubility with the pressure increasing from 8.06 to 14.82 MPa. The solubility values are very similar from 313.15 and 323.15 K at 9.30 MPa; however, an increase in solubility is observed at a temperature of 323.15 K with the same pressure, and it is considered that these are the best solubility conditions for pure perezone. The solubility isotherm at 333.15 K displayed less solubility in comparison with the temperature above.

Good perezone supercritical carbon dioxide solubility is a consequence of the non-polar nature of carbon dioxide, which changes to a slight polarity due to the existence of a quadrupole moment. Supercritical carbon dioxide can be described as a hydrophobic solvent with polarity comparable to that of *n*-hexane [31,57,58], and perezone solubility has good solubility in *n*-hexane.

Taking into account the previously determined conditions, perezone extraction was developed using 20 g of milled root and exposed to the supercritical fluid for 6, 10, 12, and 24 h, labeled as scCO2-6, scCO2-10, scCO2-12, and scCO2-24, respectively. The perezone extraction yields and selectivity are shown in Figure 2 and Figure 3.

The extraction yields, Figure 2, shows at a contact time of 6 h (scCO2-6) an amount (0.567%) less than the conventional method (1.600%) was achieved. However, when the contact time increased to 10 h (scCO2-10), the perezone yields also increased obtaining values slightly larger than the conventional heat treatment (1.817%). The treatments scCO2-12 and scCO2-24 h provided the best yields of perezone (2.243% and 2.495%, respectively) in comparison with the MH using *n*-hexane as the solvent.

Regarding the selectivity (Figure 3) of the extraction with supercritical dioxide, the treatment at scCO2-6 had a value (19.507 ± 2.985%) statically equal to conventional thermal heat, though, the increase in the treatment time caused an increase in the selectivity until scCO2-12 with a value of 79.131 ± 4.771% and decreased to 70.738 ± 2.677% at scCO2-24.

The good yields obtained with the supercritical carbon dioxide is a consequence of good penetration of the supercritical fluids into solid materials [58], the nature of the compound to extract perezone being less polar, a condition previously reported as good for extraction [44] and the aforementioned similitude about the polarity of the supercritical dioxide and the *n*-hexane (Figure 5) solvent used for the conventional extraction [59].

Finally, the results of the yields and selectivity of all tested conditions are summarized in Table 1 to allow the comparison of the results of all extraction methods.

## 3. Materials and Methods

### 3.1. General

Silica gel on TLC Al foils, 1,4-dinitrobencene and CDCl_3_ were acquired from Sigma-Aldrich (St. Louis, MO, USA); *n*-hexane and ethyl acetate were purchased from JT Baker (Ecatepec, México State, México) and used without further purification.

The extractions and the presence of the product were monitored by thin-layer chromatography (TLC) in *n*-hexane/ethyl acetate (80:20) using silica gel 60-F254 coated aluminum sheets; the corresponding visualization was achieved using a 254 nm UV lamp. ^1^H and ^13^C NMR spectra were performed using a Varian Mercury-300 spectrometer (Varian, Palo Alto, CA, USA) at 300 MHz and 75 MHz for hydrogen and carbon, respectively, employing CDCl_3_ as a solvent and TMS as an internal reference to assign the chemical shifts in parts per million of the signals. 1,4-dinitrobencene was employed as an internal pattern to establish the quantification of the product. The multiplicities are reported as singlet (s), triplet (t), and multiplet (m). The EIMS and HRMS data were determined using JEOL JMS-700 MStation mass spectrometer (JEOL, Peabody, MA, USA). The melting points were determined in a Fisher-Johns apparatus (thermofisher Scientific, Waltham, MA, USA) and are uncorrected. The microwave-assisted extraction was performed using CEM Focused Microwave™ Synthesis System (CEM Corporation, Matthews, NC, USA). The near-infrared irradiation was generated using a commercial device “Flavor-Wave^®^” (1300 W/110 V/120 V-60 Hz|220 V/240 V-60 Hz) [24]. The extraction assisted by ultrasound treatment was performed by a Branson^TM^ ultrasound bath, 1510R-DTH model, at a frequency of 42 kHz +/- 66 (Branson Ultrasonics, Danbury, CT, USA). The temperature was determined, for NIR, employing an infrared thermometer (Infrared + Type K Thermometer, Extech Instruments, Sigma Aldrich 2509388-1, Sigma Aldrich, St. Louis, MO, USA); herein, the laser pointer was directed to the reaction center. The supercritical carbon dioxide solubility and extraction were carried out using a high-pressure bomb LabAlliance A-19284 (LabAlliance, State College, PA, USA) and the pressure record was made by a transductorSensotecTHE/7093-03.

### 3.2. Plant Material

The specimen of *Acourtia platyphilla* was collected in December 2015 in the north of ther Guadalupe’s mountain chain in Coacalco of Berriozabal, State of Mexico, Mexico at the coordinates: west hill 19.606675, north hill −99.097486, height 9110 feet. The specimen was identified at the Science Faculty, the Autonomous University of the State of Mexico Herbarium by the Dr. Luis Isaac Aguilera Gómez.

### 3.3. Typical Extraction Using Thermal Conditions

The perezone, a secondary metabolite, was isolated from the vegetal specimen *Acourtia platyphilla* according to literature procedures [5,8]. Three independent experiments were conducted. Five grams of dried and milled specimen roots was mixed with 30 mL of *n*-hexane and refluxed for 3 h. After this time, the extracts were filtered and dried in vacuum. The pure product was analyzed by mean ^1^H NMR (proton nuclear magnetic resonance) to determine the yield.

### 3.4. Typical Extraction Using Non-Conventional Activating Sources

Near-infrared irradiation: three independent experiments were conducted. First 5 g of the root sample was irradiated for 15 at the minimal permitted temperature (121 °C) without a solvent, after cooling, the sample was washed with *n*-hexane (30 mL) (NIR-1); for the second test (NIR-2), the specimen roots (5 g) was irradiated for 15 min at 121 °C with 30 mL of *n*-hexane as the solvent. In this step, three 5 min irradiations were carried out, with an interval of 5 min without irradiation between each irradiation, to avoid projection of the solvent by boiling. Finally, the last test (NIR-3), 5 g of the roots sample was irradiated for 30 min with 30 mL of *n*-hexane as the solvent. It is important to mention that the irradiation was in increments of 5 min with increments of 5 min without irradiation between. After the treatments, the extracts were filtered and dried in a vacuum. The pure product was analyzed by mean ^1^H NMR to determine the yield.

Microwave irradiation: three independent experiments were conducted. For this method, three different experiments were developed. The first test (MWE-1) was carried out employing 5 g of the specimen roots with 100 W of power, for 5 min at 50 °C and 30 mL of *n*-hexane as the solvent. For the second test (MWE-2), the root sample (5 g) was exposed to 30 W of power, 50 °C, 3.5 min and 30 mL of *n*-hexane as experimental conditions, and finally for last test (MWE-3), employed 100 W, 60 °C, 10 min with 30 mL of *n*-hexane as the solvent for 5 g of specimen roots. After the treatments, the extracts were filtered and dried in a vacuum. The pure product was analyzed by mean ^1^H NMR to determine the yield.

Ultrasound extraction: three independent experiments were conducted. For the first test (US-1), 5 g of the roots sample was exposed for 30 min at 60 °C and 30 mL of *n*-hexane with a frequency of 42 kHz. For the second test (US-2) with the same temperature, solvent and frequency conditions, 5 g of the specimen roots were exposed for 60 min. After the treatments, the extracts were filtered and dried in a vacuum. The pure product was analyzed by mean ^1^H NMR to determine the yield.

### 3.5. Perezone Extraction with Supercritical Carbon Dioxide

#### 3.5.1. Perezone Solubility in scCO_2_

To propose an extraction process of perezone from the root of the plant called “pipitzahuac” using supercritical carbon dioxide, it is first necessary to conduct a study of the behavior of the thermodynamic equilibrium and kinetics of the extraction process of a robust system—fluid handling and the experimental conditions of pressure and temperature.

To find the best conditions to extract perezone, the extraction and solubility of this compound in supercritical carbon dioxide at different pressure conditions (8.06 to 14.81 MPa) and temperature (313.15–333.15 K) was determined by a static method [26,27].

**Figure 6:**
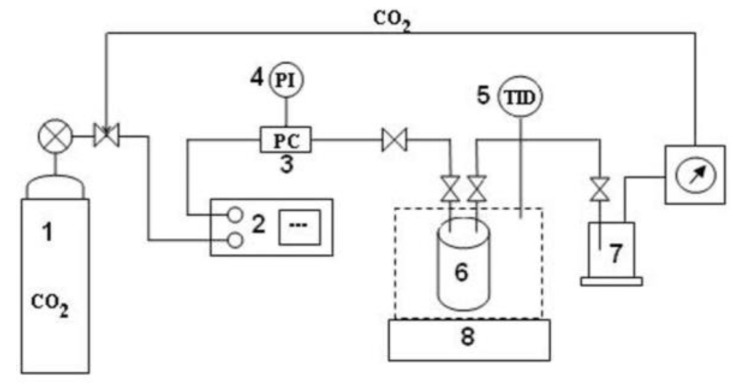
The experimental conditions tested for the solubility with pressures of 8.06, 9.30, 10.65, and 14.81 MPa with temperatures of 313.15, 323.15, and 333.15 K.

Figure 6 shows the schematic representation of the used equipment in the solubility determination. The typical experiments were developed using 0.1 g of pure perezone placed in a 3 cm^3^ solubility cell with a porous cover. This cell was introduced into the high-pressure cell (6), and the experimental temperature was achieved using a hot air chamber (8). After the initial system purge with CO_2_, the high-pressure cell was pressurized with a high-pressure bomb (2), and the pressure was recorded with the transductor (3). When the temperature and pressure were stabilized, the perezone was maintained in contact with the supercritical dioxide for 4 h. After this time, the dissolved perezone was transferred to the expansion cell (7). The solubilized perezone was determined by gravimetric determination of the perezone recovered in the expansion cell, the undissolved and original mass.

#### 3.5.2. Perezone Extraction with scCO_2_

The typical extraction experiments were performed using the equipment of Figure 6. Twenty grams of dried and milled vegetal specimen *Acourtia platyphilla* roots were placed in the high-pressure cells and extracted with scCO_2_ using the best solubility conditions, previously determined. The contact times between the vegetal material and supercritical fluid were 6, 10, 12, and 24 h. The pure product was analyzed by mean ^1^H NMR to determine the yield.

#### 3.5.3. Perezone Quantification in the Extract

**Figure 7:**
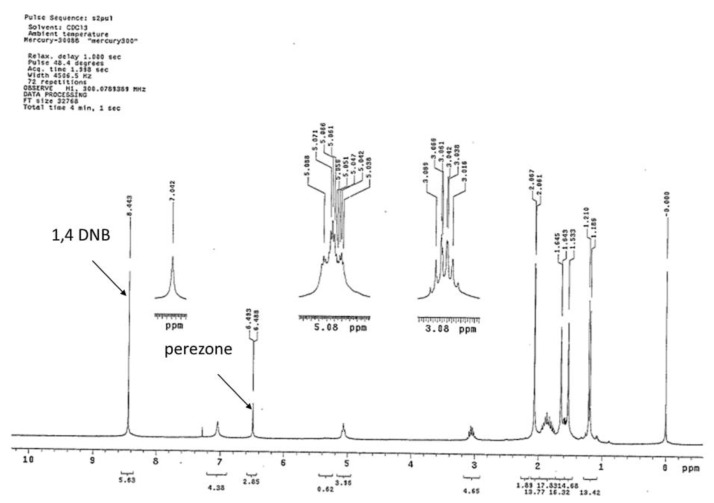
^1^HNMR of perezone extract with the 1,4 DNB as standard.

The perezone quantification in the extracts was made by ^1^H NMR determinations [60,61,62,63], using as internal standard 1,4-dinitrobencene (1,4-DNB). The analysis sample was prepared by the mix of 30 mg of perezone extract with 10 mg of 1,4-DNB, dissolving the mixture in 0.7 mL of CDCl_3._ The perezone quantity of the samples was obtained by Equation (1):

(1) $$Cperezone=\frac{I_{p}}{I_{std}}\times \frac{N_{std}}{N_{p}}\times \frac{Mw_{p}}{Mw_{std}}\times C_{std}$$

where *I*, *N*, *Mw*, and *C* are the integral areas, number of nuclei, molecular weight, and concentration, respectively, for the perezone (p) and the internal standard (std). From this value, two parameters were obtained: Selectivity considered as the perezone contents in the extract, and yield considered the perezone percentage in the analyzed root. See Figure 7 as an example.

### 3.6. Statistical Process Control

Analysis of variance was performed using the ANOVA procedure. Tukey’s test was used to determine the differences of means, and the results were considered statistically significant at *p* < 0.05. Data analysis was carried out using Stat graphics Centurion XVI software, version 16.2.04 (64 bits) 1982–2013 (StatPoint Technologies, Inc., Warrenton, Va, USA).

### 3.7. Perezone Spectral Data

^1^H NMR (300 MHz, CDCl_3_): δ 1.06 (d, 3H), 1.53 (s, 3H), 1.64 (s, 3H), 1.69 (m, 2H), 1.89 (m, 2H), 2.06 (s, 3H), 3.03 (m, 1H), 5.04 (t, 1H), 6.47 (s, 1H), 7.21 (s, OH). ^13^C NMR (70 MHz, CDCl_3_): δ 14.86, 17.59, 18.21, 22.34, 26.44, 29.29, 34.08, 124.43, 124.44, 127.39, 135.84, 143.47, 144.63, 194.01, 203.97. EIMS (70 eV) *m/z* (% ar): 249 (20%) [M+1]^+^, 248 (20%) M^+.^, 231 (6) [M-17]^+^, 166 (100) [M-82]^+^.

## 4. Conclusions

A comparative study of the extraction of perezone from *Acourtia platyphilla* using non-conventional activation sources was developed, observing, in the most cases, best yield, best selectivity, and less extraction time in comparison with conventional treatment. In addition, the perezone solubility study in supercritical dioxide, and the extraction of the milled roots was achieved. Taking into account the obtained results, it can be concluded that near-infrared and ultrasound-assisted extractions gave the best yields. However, infrared-assisted extractions required less time. Regarding supercritical carbon dioxide extractions, these had similar yields compared to microwave-assisted ones, despite the former having better selectivity, yielding neater perezone than the latter.

The methodologies employed were in agreement with the following green chemistry principles: safer solvents and auxiliaries (Principle 5) and design for energy efficiency (Principle 6).

## Figures and Tables

**Table 1:** Results of the extraction processes for perezone.

Methods	Yield (%)	Standard Deviation
MH ^1^	1.652	0.119
NIR-1 ^2^	1.656	0.027
NIR-2 ^3^	2.036	0.043
NIR-3 ^4^	6.271	0.554
MWE-1 ^5^	1.027	0.026
MWE-2 ^6^	1.353	0.065
MWE-3 ^7^	2.996	0.008
US-1 ^8^	5.609	0.322
US-2 ^9^	6.623	0.322
scCO2-6 ^10^	0.564	0.018
scCO2-10 ^11^	1.818	0.045
scCO2-12 ^12^	2.243	0.046
scCO2-24 ^13^	2.495	0.043

^1^ Samples refluxed for 3 h with solvent. ^2^ Samples irradiated without solvent. ^3^ Samples irradiated for 15 min with solvent. ^4^ Samples irradiated for 30 min with solvent. ^5^ Samples irradiated for 5 min, 100 W, and 50 °C with solvent. ^6^ Samples irradiated for 3.5 min, 30 W, and 50 °C with solvent. ^7^ Samples irradiated for 10 min, 100 W, and 60 °C with solvent. ^8^ Samples sonicated for 30 min, and 60 °C with solvent. ^9^ Samples sonicated for 60 min, and 60 °C with solvent. ^10^ Samples extracted with scCO_2_ for 6 h at 50°C, and 10.65 MPa. ^11^ Samples extracted with scCO_2_ for 10 h at 50 °C, and 10.65 MPa. ^12^ Samples extracted with scCO_2_ for 12 h at 50 °C, and 10.65 MPa. ^13^ Samples extracted with scCO_2_ for 24 h at 50 °C, and 10.65 MPa. See Materials and Methods section.

## Abbreviations

FIR	Far infrared
^1^H NMR	Proton nuclear magnetic resonance
IR	Infrared
MH	Mantle heating
MIR	Middle infrared
MM	Tribochemistry-mechanical milling
MW	Microwave
MWE	Microwave extraction
NIR	Near-infrared
scCO_2_	Supercritical carbon dioxide
scCO2-6	Supercritical carbon dioxide extraction at 6 h
scCO2-10	Supercritical carbon dioxide extraction at 10 h
scCO2-12	Supercritical carbon dioxide extraction at 12 h
scCO2-24	Supercritical carbon dioxide extraction at 24 h
US	Ultrasound
